# Composition Changes in *Lycium ruthenicum* Fruit Dried by Different Methods

**DOI:** 10.3389/fnut.2021.737521

**Published:** 2021-10-05

**Authors:** Youyuan Lu, Xiangfeng Kong, Juanhong Zhang, Chao Guo, Zhuo Qu, Ling Jin, Hanqing Wang

**Affiliations:** ^1^College of Pharmacy, Ningxia Medical University, Yinchuan, China; ^2^Ningxia Engineering and Technology Research Center for Modernization of Regional Characteristic Traditional Chinese Medicine, Ningxia Medical University, Yinchuan, China; ^3^Ningxia Super-Kernel Health Management Technology Co., Ltd, Yinchuan, China; ^4^School of Pharmacy, Gansu University of Chinese Medicine, Lanzhou, China; ^5^Northwest Collaborative Innovation Center for Traditional Chinese Medicine, Lanzhou, China; ^6^Key Laboratory of Hui Ethnic Medicine Modernization, Ministry of Education, Ningxia Medical University, Yinchuan, China

**Keywords:** *Lycium ruthenicum*, drying methods, phytochemical content, antioxidant activity, appearance characteristic

## Abstract

The fruit of *Lycium ruthenicum* (LRF), known as black wolfberry, is a medicinal and edible fruit. The fresh LRF is perishable and has only about 3 days of shelf life. Drying could prolong the shelf life of LRF. However, it could imply physical changes and chemical modification. This study evaluated the effect of sun drying (SD), hot air drying (HD), and freeze drying (FD) on the appearance characteristics, moisture content, bioactive compounds, amino acid composition, and antioxidant activity of LRF. The results showed that LRF dried by FD was round, expansive, fragile, and maintained the largest amount of appearance traits among the three drying methods. Drying methods had a significant effect on phytochemical content and antioxidant activity of LRF (*P* < 0.05). Principal component analysis (PCA) showed that procyanidin content (PAC), asparagine (Asn), total phenolic content (TPC), total anthocyanin content (TAC), and moisture content were the main sources of the difference in LRF dried by different methods. The characteristic of LRF in FD was low moisture content, and high TPC, Asn, PAC, and TAC. Sun drying was opposite to FD. Hot air drying was high TPC and low TAC content. The quality of LRF was in the order of FD > HD > SD based on comprehensive evaluation of the phytochemical component content and antioxidant capacity. Additionally, the water temperature and soaking time had different antioxidant activity effect on LRF dried by different methods. These findings will provide useful information for production and utilization of LRF.

## Introduction

*Lycium ruthenicum* (LRF) is an ideal plant for alleviating the degree of soil salinity and alkalinity in northwest China and plays an important role in the restoration of desert ecosystems (1). The fruit of LRF, known as black wolfberry, is a medicinal and edible fruit. It has a high concentration of functional and nutritional components, such as anthocyanins, phenolic acids, flavonoids, sugars, fatty acids, and alkaloids (1–3). In LRF, it contains more than 10% polysaccharides, about 11% protein, abundant unsaturated fatty acids, minerals including macroelements such as sodium, magnesium, and iron and microelements such as manganese, zinc, and chromium (4). Anthocyanins are the main active ingredient in LRF, and with a wide range of biological activity such as antioxidant (5), anti-Alzheimer (6), anti-inflammatory (7), neuroprotective effects (8), and so on. Polysaccharides in LRF can be immune enhancing (9), anti-diabetic (4), anti-inflammatory (10), and so on. Phenolic compounds as another active ingredient could scavenge free radicals and have neuroprotective effects (11). Additionally, amino acids are important in the field of food science and nutrition. For example, essential amino acids must be consumed from the diet; free amino acid can contribute to the flavor and test of many foods; aromatic amino acids phenylalanine and tyrosine, and heterocyclic amino acid tryptophan are best for the nervous system (12).

Used as medicine, LRF has a great influence on the development of minority medicine (1, 13); and used as nutritional food, it has been eaten as fruit or used as raw materials for beverages (1, 14, 15). However, like the fruit of *L. barbarum*, fresh LRF is perishable, and has only about 3 days of shelf life when fresh (16). Drying could reduce water activity, prolong shelf life, and remain the nutrients (17). It is usually used to preserve and store food products (18). Thus, dry fruit is the main source of LRF for medicinal and edible use. Drying can be done in many ways. Sun drying (SD) is the conventional drying technique for drying agricultural products (19, 20). With the development of science and technology, hot air drying (HD) and freeze drying (FD) techniques have been applied for fruits, vegetables, and herbs (21). It is indicated that drying could imply physical changes and chemical modifications (18). Chemical modifications include color changes, flavor losses, bioactive and nutritional compounds degradation, and antioxidant activity reducing, and could be influenced by various drying techniques (20, 22, 23). For example, freeze drying preserved more than 97% of L-ascorbic acid in raspberry, while convective drying samples had degradation of over 80% of this compound (24). The freeze drying strawberry powders were characterized by the highest content of vitamin C and polyphenols, while the content of these ingredients in convective drying and spray drying powders was lower by 55–80% for vitamin C and 80% for the polyphenols content. In addition, the flavor was most beneficial for the freeze drying powders (25). Based on the above studies and the instability of some chemical compounds in LRF, such as anthocyanins which are susceptible to pH, light, temperatures, and metal ions (26), polyphenols have structures that are very easily oxidized, which can lead to degradation of antioxidant capacities (27) and the thermal stabilities of amino acids are much different (28). Thus, to preserve the bioactive compounds and nutritional components, a better drying method is required (29). However, thus far, there is little relevant research reported about the effects of the drying process on the quality of LRF.

In the present study, the moisture content, bioactive compounds, amino acid composition, and antioxidant activity of LRF treated by three drying methods (SD, HD, and FD) were assessed. Meanwhile, soaking time and water temperature for the antioxidant activity effect of LRF with the three drying methods were investigated. The purposes of this study: (1) illustrated the characteristics of the three drying methods used for LRF; (2) screened a drying method that can maximize preservation of the components in LRF; and (3) provided guidance for soaking edible LRF with higher values. These findings will provide useful information for production and utilization of LRF.

## Materials and Methods

### Chemicals and Reagents

Reference compounds of gallic acid (GA), procyanidins (PA), cyanidin 3-glucoside (CG0), rutin, proline (Pro), leucine (Leu), arginine (Arg), asparagine (Asn), aspartic acid (Asp), lysine (Lys), tyrosine (Tyr), glutamine (Gln), phenylalanine (Phe), glutamic acid (Glu), threonine (Thr), serine (Ser), iso-leucine (Ile), histidine (His), ornithine hydrochloride (Orn), valine (Val), citrulline (Cit), methionine (Met), and tryptophan (Try) were purchased from Sigma-Aldrich (St. Louis, MO). The purity of each compound was more than 98%, determined by HPLC analysis. The reagents of 2,2-diphenyl-1-picrylhydrazyl (DPPH), 2,4,6-tripyridyl-S-triazine (TPTZ), and Folin-Ciocalteu phenol reagent were purchased from Linen Technology Development Co., Ltd. (Shanghai, China). HPLC-grade acetonitrile and formic acid were obtained from Merck (Darmstadt, Germany). Ammonium formate and ammonium acetate (mass spectrum grade) were acquired from Shanghai Chemical Reagent Factory (Shanghai, China). Deionized water was prepared by a Milli-Q water purification system (Millipore, MA). Other reagents and chemicals were analytical grade.

### Plant Identification and Sample Preparation

The ripe fresh LRF samples were collected from Ningxia Hui Autonomous Region of China in July 2020. The botanical origins were identified as *Lycium ruthenicum* L. by Prof. Hanqing Wang. The LRF surface is covered with a waxy layer. During the drying process, the waxy layer will block moisture removal from the material, and then affect the drying effect (16). Drying combined with some pretreatments is a cost-effective method of preservation (30). Thus, the samples were rinsed with a 3%0 NaH_2_CO_3_ solution to pretreatment, then washed with water. Then, 500 g of samples was dried by each of the test methods, and each of the test methods was done in triplicate.

- For SD, samples were spread on trays with a single layer and placed in the dark for 2 h. Then, the drying trays were exposed to the sun at 20–32°C until the epidermis wrinkled. Finally, the drying trays were placed in the shade for approximately 10 days.
- For HD, the samples were placed in a ventilated oven (Faraz Electric, Iran) at 40°C for 7 h. Then, they were moved to a natural environment for 7 h before being placed in a drying oven at 45°C for 4 h. Finally, the samples were maintained at 50°C for 12 h and then at 60°C for 5 h.
- For FD, LRF was first frozen in a freeze dryer (JDG-0.2, Lanzhou Kejin Freeze-Drying Instruments Co., Ltd., Lanzhou, China). Then, the temperature was gradually lowered to −30°C, and the vacuum was continuously increased to 50 Pa. Finally, the temperature was gradually lowered to −60°C and maintained for 30 h.

After each drying process, the samples were ground into a fine powder (40 mesh) and stored at 4°C for further analysis.

### Moisture Content

A sample (2 g) was weighed into aluminum cans for moisture content determination in an oven (Faraz Electric, Iran) at 100–105°C until constant weight (31).

### Sample Extraction of Phenolic-Antioxidant Compounds

The method of ethanol extraction was performed according to the method of Zhang et al. (32) with slight modification. A total of 0.1 g LRF powders (40 mesh) was extracted with 15 ml 80% ethanol in 50°C ultrasonic bath (40 kHZ) for 60 min. The extract was followed by centrifugation at 15,000 × *g* for 5 min, and the supernatant was separated for analysis of phenolic, flavonoids, procyanidin, anthocyanin, and antioxidant activity.

The method of water extraction was performed as follows: a total of 0.2 g LRF powders (40 mesh) was extracted with 50 ml water at different temperatures (0, 20, 40, 60, 80, and 100°C) for different times (5, 10, 30, 45, 60, 180, 360, and 600 min), respectively. Then, the supernatant was separated for antioxidant activity assay.

### Total Phenolic Content

Total phenolic content (TPC) was measured according to the Folin-Ciocalteu assay (33) with slight modification. Briefly, 0.2 ml sample solution was diluted in 4 ml of distilled water and then mixed with 0.4 ml of the Folin-Ciocalteu reagent for 3 min before 1.8 ml of 20% Na_2_CO_3_ solution was added to the mixture and finally incubated for 2 h in the dark at room temperature. The absorbance of the solution was measured at a wavelength of 765 nm. Results were expressed as mg gallic acid equivalents per g dry weight (mg GAE/g DW).

### Total Flavonoid Content

Total flavonoid content (TFC) was measured according to an Al(NO_3_)_3_ colorimetric method described by Yang et al. (33) slightly modified. In brief, 0.3 ml of 5% NaNO_2_ was added to 1 ml of prepared sample solution, and the mixture was allowed to stand for 5 min. Then, 0.3 ml of 10% Al(NO_3_)_3_ was added to the mixture, followed by adding 4 ml of 1 mol/L NaOH after 6 min. Subsequently, the solution was brought up to 10 ml with 80% ethanol. The mixture was left to stand for 30 min at room temperature, and the absorbance was measured at 510 nm. Results were expressed as mg rutin equivalents per g dry weight (mg RE/g DW).

### Procyanidin Content

Procyanidin content (PAC) was measured using the butanol-HCl method (34). The chemicals first added to the 1 ml sample solution, 6 ml butanol-HCl reagent which was made from the mixing of butanol and HCl with the ratio of 19:1(v/v), and 0.2 ml of 2% (w/v) ammonium ferric sulfate. Subsequently, the mixture was mixed and put in a boiling water bath for 60 min. After that, the temperature of the mixture was decreased at room temperature. The absorbance was measured at 550 nm. Results were expressed as mg procyanidin equivalents per g dry weight (mg PAE/g DW).

### Total Anthocyanin Content

Total anthocyanin content (TAC) was measured using a pH-differential method describe by Wang et al. (35). A 3-ml sample solution was mixed with 7 ml 0.025 mol/L potassium chloride buffer (pH 1.0), and the other 3-ml sample solution was mixed with 0.4 mol/L sodium acetate buffer (pH 4.5). The mixture was left to stand for 90 min at room temperature, and the absorbance was measured at 520 and 700 nm for both solutions, respectively. Results were expressed as mg cyanidin 3-glucoside equivalents per g dry weight (mg CGE/g DW).

### Individual Amino Acid Content

A total of 0.25 g LRF powders (40 mesh) was extracted with 50 ml water and ultrasonic bath (40 kHz) for 30 min at room temperature. Additional water was added to compensate for weight loss during the extraction. Then, the samples were filtered through 0.22-μm membrane filters prior to injection in the UHPLC system.

The parameters of hydrophilic interaction chromatographic separation and mass spectrometry for amino acid analysis were employed according to Wang et al. (36) with some modification. The mobile phase was composed of A (0.8% acetic acid, 10 mmol/L ammonium acetate in deionized water) and B (0.1% acetic acid in acetonitrile in acetonitrile) with a gradient elution (0–2 min, 10% A; 2–5 min, 10–25% A; 5–8 min, 25–40% A; 8–10 min, 40–50% A; 10–12 min, 50–90% A). The flow rate of the mobile phase was 0.25 ml/min, and the column temperature was maintained at 20°C. The injection volume was 1 μl. Mass spectrometry was performed using an electrospray ionization (ESI) source operated in positive ion mode and the parameters were set as follows: the capillary voltage at 3 kV, the desolvation gas flow rate set to 1,000 L/h and temperature at 550°C, the cone gas flow rate at 50 L/h at a temperature of 150°C. The cone voltage and collision energy were set to match the multiple reaction monitoring (MRM) of each marker.

### DPPH Free-Radical Scavenging Assay

The DPPH free radical scavenging activity of the extracts was used based on the procedure described by Yang et al. (33) with some modification. Aliquots of each extract (0.2 ml) were added to 4.8 ml of ethanolic DPPH solutions (0.1 mM). The mixture was mixed and stood in the dark for 30 min at room temperature. The absorbance was read at 517 nm. The antioxidant activity of 80% ethanol extract was expressed as IC_50_ (the antioxidant concentration required to reduce the DPPH absorbance by half), and the antioxidant activity of the water extract was shown as % loss (37).

### Ferric Reducing/Antioxidant Power Assay

The Ferric reducing/antioxidant power assay (FRAP) assay was performed according to Yang et al. (33) described with slight modification. A 0.1-ml sample solution and 2-ml distilled water were added to 3 ml of FRAP reagent. The absorbance was recorded at 593 nm after incubation for 50 min in the dark at 37°C with shaking. The activity was expressed as mmol Fe^2+^/g DW.

### Statistical Analysis

All results were expressed as mean ± standard deviation (SD) of three replicates. Data were analyzed by one-way analysis of variance (one-way ANOVA), along with Duncan's test. Statistical analysis was performed using SPSS 19.0 (SPSS Inc., Chicago, IL). Principal component analysis (PCA) was performed using SIMCA-P 14.1 (Umetrics Inc., Sweden). An antioxidant contour map of the water extract was performed using graphing software (OriginPro 8.0, Northampton, MA).

## Results and Discussion

### Effect of Drying Methods on the Appearance Quality of LRF

**Figure 1:**
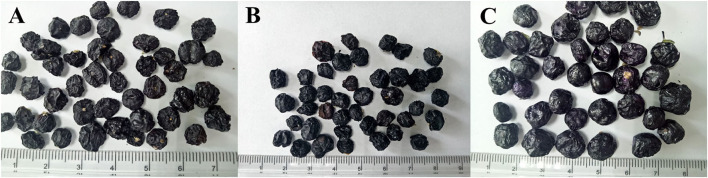
The appearance traits of the *Lycium ruthenicum* fruit (LRF) dried by different methods. (**A**: sun drying, SD; **B**: hot air drying, HD; **C**: freeze drying, FD).

Appearance quality reflects the ability to attract consumers, and is the main factor affecting the market value of products (38, 39). Drying is the best method to preserve fruits. Meanwhile, it could lead to a difference on the appearance quality of fruits (21). The appearance traits of LRF dried by different methods were shown in Figure 1. The color of LRF dried by SD and HD was dark purple, and that dried by FD was bright purple. For the shape and structure, LRF dried by FD was round. The fruit was expansion and fragile. However, the fruit dried by SD and HD was shrinkage and hardness, especially for the fruit dried by SD. Drying of food involves complicated processes related to the mass, heat, and momentum transport. Therefore, structure change has a relationship with moisture content and is relevant to the different heating transfer modes (40). In general, lowering pressure and shortening drying time helped to prevent structure collapse of foods during drying (41). Sun drying promoted a long time, followed by HD, which was explained by the fact that a long drying time leads to the product shrinking. In addition, for FD, the drying shrinkage could be inhibited by the lower air pressure. Thus, LRF dried by FD showed a less collapsed structure and maintained a larger amount of appearance traits, followed by LRF drying using HD.

### Effect of Drying Methods on Moisture Content of LRF

**Figure 2:**
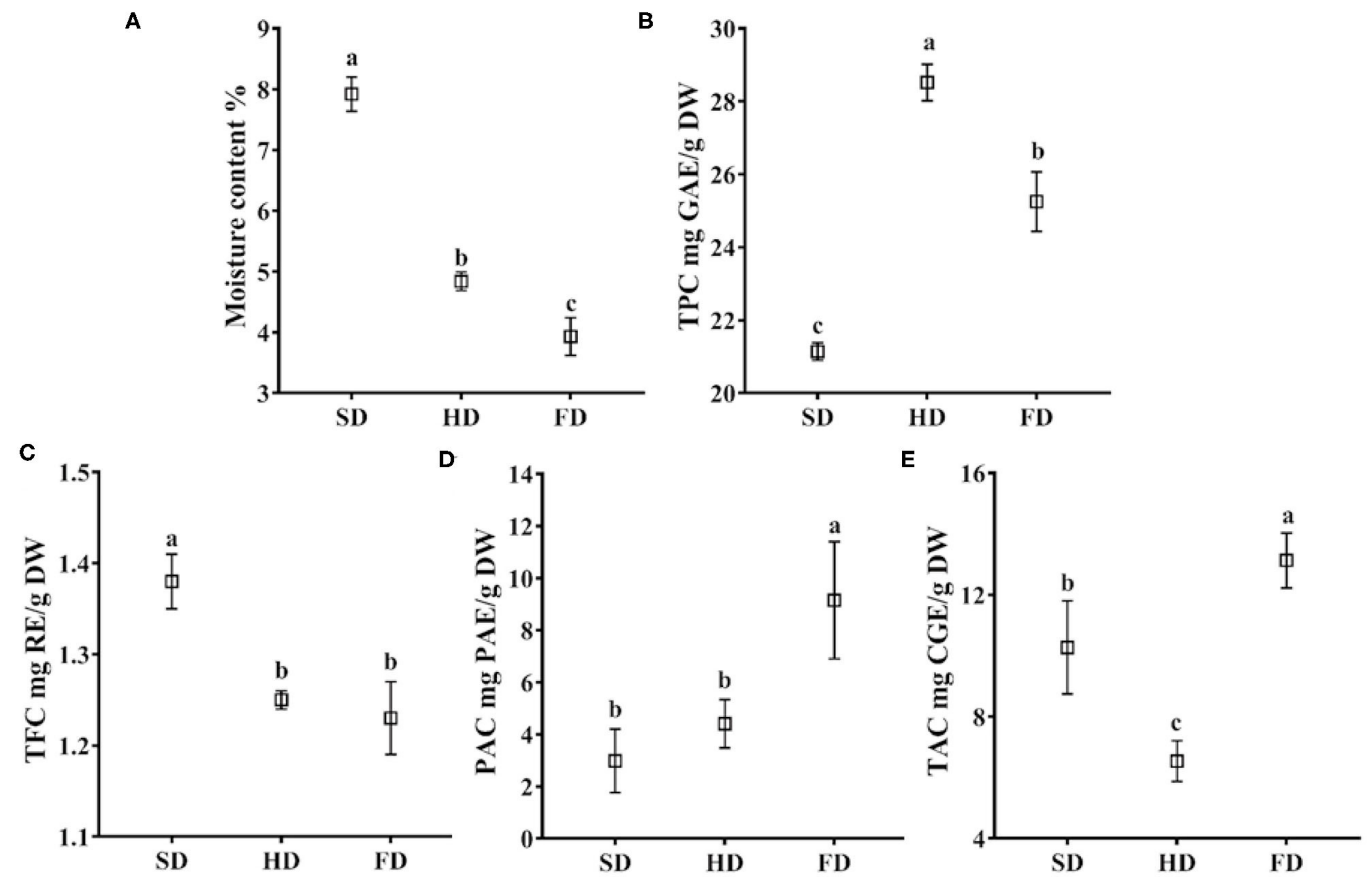
The moisture content **(A)**, total phenolic content **(B)**, total flavonoid content **(C)**, procyanidin content **(D)**, and total anthocyanin content **(E)** in LRF dried by SD, HD, and FD. The same lowercase letters above the values for the same component are not significantly different (*p* > 0.05).

Drying is aimed to be a reduction in water activity of the solid product by removing the majority of the water content. Thus, water activity was the easiest parameter to evaluate the drying method (18). The moisture content of LRF dried by different methods was significantly different (*P* < 0.05, Figure 2A). The moisture content of LRF dried by FD was the lowest (3.93%), followed by HD (4.84%) and SD (7.92%), respectively. It may be explained by the characteristics of the different drying methods. For the FD process, moisture was directly converted from the solid state to a gaseous state without passing through an intermediate liquid phase. It could prevent structure collapse of the product, remove more moisture, and finally with good rehydration capacity (21, 22, 40). For the SD process, moisture was evaporated at a slow rate, and the samples always shrink. It can lead to undesired moisture remaining trapped in the LRF. Compared to SD, HD had higher temperatures. It could increase the moisture removed from LRF.

### Effect of Drying Methods on TPC of LRF

Drying methods had significant effects on the TPC of LRF (*P* < 0.05, Figure 2B). The highest was found in HD (28.52 mg GAE/g DW), followed by FD and SD (25.25 and 21.14 mg GAE/g DW, respectively). It was consistent with the Li et al. (42) research that HD was recommended as a beneficial drying method for preserving the phenolic content. These findings may be explained by oxidative reaction. Hot air drying could inhibit enzymatic oxidative reaction to some extent for the higher temperature (42). During SD, both non-enzymatic and enzymatic oxidative reaction are likely taking place. While for the lower exposure to oxygen of FD, the enzymatic oxidative reaction by polyphenol oxidase and peroxidase is more likely to occur. In addition, the damage to the cell structure caused by ice crystal formation could promote a loss in the content of phenolic compounds (43).

### Effect of Drying Methods on TFC of LRF

The results of TFC in LRF dried by three different methods were shown in Figure 2C. The TFC of LRF after drying by different methods, from high to low, was: SD (1.38 mg RE/g DW) >HD (1.25 mg RE/g DW) >FD (1.23 mg RE/g DW). There was no significant difference (*P* > 0.05) of TFC in HD and FD. Compared to HD, SD was performed at low temperature. It could prevent the loss of a heat-sensitive compound. Additionally, the drying method has a slow drying rate, which could keep the metabolic process to continue after harvest (44). All those may be the reason that TFC in LRF of SD was the highest among the three drying methods.

### Effect of Drying Methods on PAC of LRF

Procyanidins is widely distributed in fruits, juice, wine, and tea. It contributes to the bitter flavor and astringency, and impacts the mouth feel (45). Drying methods had significant effects on the PAC of LRF (*P* < 0.05, Figure 2D). The PAC of LRF dried by three methods ranged from 2.98 to 9.15 mg PAE/g DW, and from high to low was FD > HD > SD. Freezing and cold storage have relatively little impact on procyanidin, and high temperature could lead to a profound reduction in procyanidin (46). The temperatures of SD and HD were both higher than FD. Thus, temperature may be the reason that caused PAC to be significantly different in LRF dried by the three methods.

### Effect of Drying Methods on TAC of LRF

Anthocyanin is responsible for the purple color of LRF and is the abundant and the main active ingredient of this species (35). Anthocyanin is susceptible to temperature, oxygen, and light (26). During drying, all of these factors are unpredictable, and ultimately affect TAC of the product (*P* < 0.05). The results of TAC of LRF dried by three methods are shown in Figure 2E. The highest was found in FD (13.13 mg CGE/g DW), followed by SD (10.27 mg CGE/g DW). The TAC in HD was only 6.54 mg CGE/g DW. The results were consistent with Nemzer et al. (47) reporting that FD products exhibited a better retention of anthocyanin than HD fruits. Anthocyanin is degraded by high temperature (23), which may be the reason that HD had the lowest TAC among the three drying methods.

It is reported that during the thermal degradation process of anthocyanin, the anthocyanin glycosides were generally cleaved by successive loss of sugar moieties, and then anthocyanin aglycones were further degraded by scission into phloroglucinaldehyde (cyanidin, pelargonidin), 4-hydroxybenzoic acid (pelargonidin), and protocatechuic acid (cyanidin), the residues of the A- and B-rings, respectively (48). Thus, it may be the reason for the decrease of anthocyanin in HD and SD. Additionally, compared to the bioactive components content in this study, the highest TPC and the lowest TAC were observed in HD, which may be illustrated by the result that the anthocyanin degradation could produce phenolic acids (49).

### Effect of Drying Methods on Amino Acid of LRF

Based on previous study results (36), a hydrophilic interaction chromatography ultra-performance liquid chromatography coupled with triple-quadrupole tandem mass spectrometry (HILIC-UHPLC-TQ-MS) method was used to analyze amino acids in LRF. The precursor/product ion pairs and parameters for MRM of the analyte used in the study are shown in Supplementary Table 1. The typical chromatograms of the standards are presented in Supplementary Figure 1. The method validation was evaluated according to the linearity, limit of detection (LOD) and quantitation (LOQ), intra-day and inter-day precisions, and stability and accuracy, and the results are shown in Supplementary Table 2. Each standard composition showed good linearity (*r*^2^ > 0.995). The LOD and LOQ-values were in the range of 5.30–16.20 and 14.00–32.50 ng/ml, respectively. The intra-day, inter-day, repeatability and stability variations (RSD)-values of these compounds were <5.64%. The recovery ranged from 94.10 to 102.80%, and the RSD ranged from 2.32 to 5.54%. All of these results illustrated that the method was sensitive, repeatable, and accurate for quantification of these amino acids.

A total of 19 amino acids were determined in LRF based on the developed method, including eight essential amino acids and two semi-essential amino acids (Arg and His). The contents of 19 amino acids in LRF dried by three methods are summarized in Table 1. Asn and Arg showed higher amounts in LRF, followed by Gln, Asp, and Ser. Drying methods have significant effects on the content of amino acids in LRF (*P* < 0.05). The content of total amino acids ranged from 1.6471 to 7.3750 mg/g, and from high to low was FD > HD > SD. The individual amino acid content is the highest in FD expect for Pro, which is the lowest in FD. The content of Asp, Tyr, and Orn was no significant difference between SD and HD, others were shown that HD was higher than SD. It is worth noting that Ser, Ile, and Met were not detected in SD.

**Table 1:** Contents of amino acids (mg/g) in *Lycium ruthenicum* fruit (LRF) dried by different methods.

**Drying method**	**SD^a^**	**HD**	**FD**
Pro^b^	0.2245 ± 0.0012b	0.2383 ± 0.0032a	0.2064 ± 0.0002c
Leu	0.0620 ± 0.0006c	0.0789 ± 0.0001b	0.1371 ± 0.0007a
Arg	0.4824 ± 0.0014c	0.5868 ± 0.0025b	1.2690 ± 0.0030a
Asn	0.0959 ± 0.0027c	1.0989 ± 0.0066b	2.8499 ± 0.0113a
Asp	0.2069 ± 0.0105b	0.2163 ± 0.0033b	0.3949 ± 0.0016a
Lys	0.0597 ± 0.0018c	0.0681 ± 0.0004b	0.1038 ± 0.0004a
Tyr	0.0521 ± 0.0017b	0.0517 ± 0.0001b	0.1370 ± 0.0010a
Gln	0.1370 ± 0.0014c	0.1618 ± 0.0008b	0.6210 ± 0.0014a
Phe	0.0060 ± 0.0001c	0.0661 ± 0.0009b	0.1686 ± 0.0011a
Glu	0.0977 ± 0.0017c	0.1041 ± 0.0001b	0.1875 ± 0.0027a
Thr	0.0285 ± 0.0016c	0.0625 ± 0.0051b	0.1806 ± 0.0035a
Ser	Nd^c^	0.1116 ± 0.0011b	0.3393 ± 0.0014a
Ile	Nd	0.0593 ± 0.0156b	0.1331 ± 0.0001a
His	0.0976 ± 0.0024c	0.1502 ± 0.0004b	0.2648 ± 0.0025a
Orn	0.0263 ± 0.0001b	0.0372 ± 0.0057b	0.0625 ± 0.0112a
Val	0.0379 ± 0.0131c	0.0872 ± 0.0183b	0.1673 ± 0.0006a
Cit	0.0090 ± 0.0008c	0.0152 ± 0.0020b	0.0545 ± 0.0019a
Met	Nd	0.0077 ± 0.0000b	0.0104 ± 0.0018a
Try	0.0235 ± 0.0020c	0.0474 ± 0.0011b	0.0875 ± 0.0040a
Total	1.6471 ± 0.0165c	3.2480 ± 0.0090b	7.3750 ± 0.0048a

a *SD, sun drying; HD, hot air drying; FD, freeze drying*.

b *proline (Pro), leucine (Leu), arginine (Arg), asparagine (Asn), aspartic acid (Asp), lysine (Lys), tyrosine (Tyr), glutamine (Gln), phenylalanine (Phe), glutamic acid (Glu), threonine (Thr), serine (Ser), iso-leucine (Ile), histidine (His), ornithine hydrochloride (Orn), valine (Val), citrulline (Cit), methionine (Met), and tryptophan (Try)*.

c *Not detected*.

*Values followed by the same lowercase letters in the same row are not significantly different (p > 0.05)*.

The results were consistent with Chumroenphat et al.'s (22) research that drying method could affect the amino acid content in product, which may be caused by Maillard reaction involved in different parameters such as water activity, time, and temperature (50). Additionally, environmental stress could cause the accumulation of Pro (51), which may be the reason that Pro content in SD and HD was higher than FD. It has reported that Try, His, Lys, Met, and Arg are the amino acids that are most affected by reactions taking place during this process (52). The study results showed that Ser and Ile were also the amino acids that were most affected by drying methods.

### Effect of Drying Methods on Antioxidant Activity of LRF

**Figure 3:**
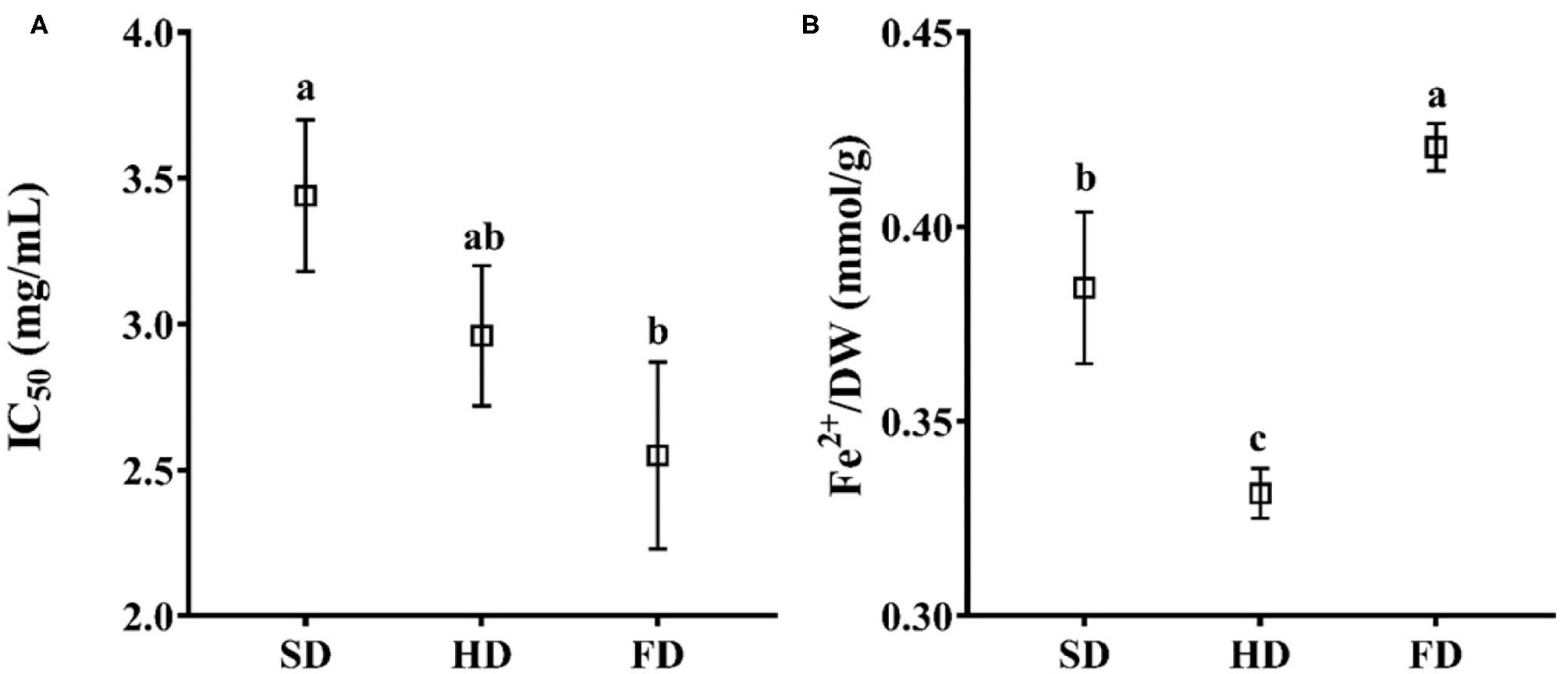
The antioxidant activity of LRF dried by SD, HD, and FD (**A**: DPPH; **B**: FRAP). The same lowercase letters above the values for the same component are not significantly different (*p* > 0.05).

The DPPH radical scavenging activities and reducing powers (FRAP) of LRF dried by different methods are shown in Figure 3. The IC_50_ of DPPH (Figure 3A) for 80% ethanol extract ranged from 2.55 to 3.44 mg/ml. The antioxidant activity of FRAP (Figure 3B) ranged from 0.3315 to 0.4205 mmol Fe^2+^/g DW. ANOVA analysis showed that the antioxidant activity of LRF dried by different methods exhibited a significant difference (*P* < 0.05) in assays of DPPH and FRAP. The highest antioxidant activity was found in FD, and DPPH IC_50_ was 2.55 mg/ml (the higher values mean the lower antioxidant capacity), and the value of FRAP was 0.4205 mmol Fe^2+^/g DW. The DPPH IC_50_ for SD (3.44 mg/ml) and HD (2.96 mg/ml) had no significant difference (*P* > 0.05), and the values of FRAP for SD (0.3843 mmol Fe^2+^/g DW) were significantly different with HD (0.3315 mmol Fe^2+^/g DW). The reason for this finding may be the induction of heat resistant antioxidant enzymes (quinone oxidoreductase and superoxide dismutase) not measured in this study (53). Additionally, the difference in phytochemicals could cause different reactions with DPPH and FRAP under different drying methods, which may contribute to this finding (54).

### Principal Component Analysis

**Figure 4:**
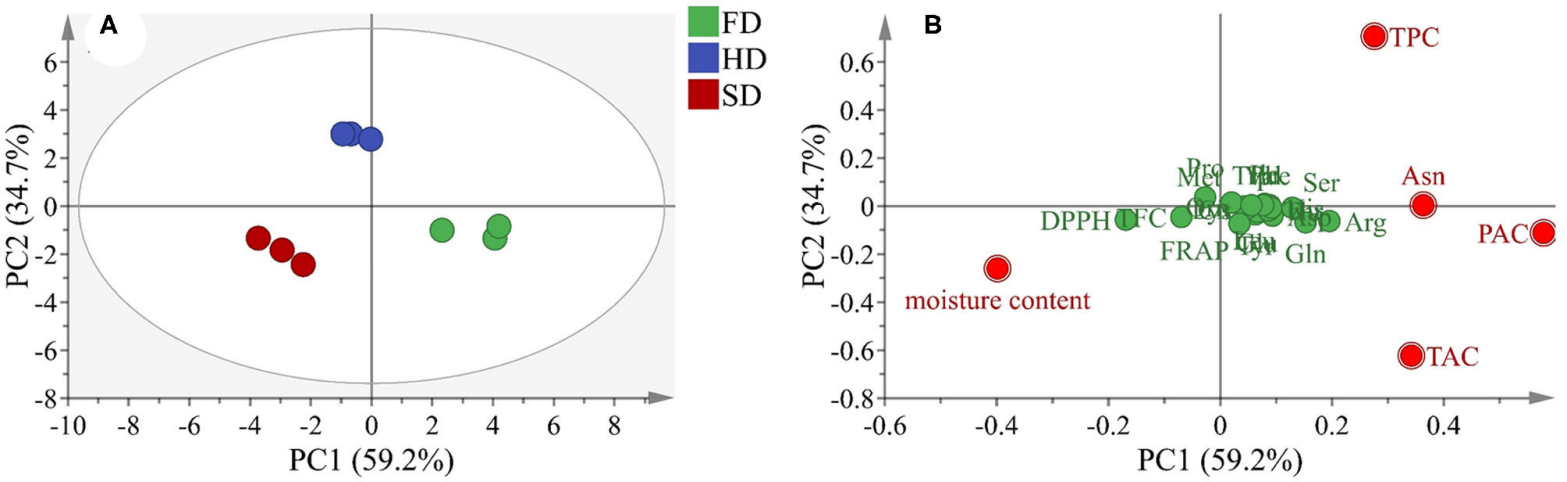
Principal component analysis (PCA) according to phytochemical content and antioxidant activity of LRF dried by SD, HD, and FD (**A**: score plot; **B**: loading scatter plot).

To further explore the variation in LRF dried by different methods, a PCA test was performed based on the quantitative analysis and antioxidant capacity, and the results are shown in Figure 4. The first two factors (PC1 and PC2) occupied up to 93.9% (PC1 represents 59.2% and PC2 represents 34.7%) of the total variation. The scatter points were clearly exhibited through the loading of the variables. The loading plot (Figure 4B) showed that PAC, Asn, TPC, TAC, and moisture content were far away from the x-axis and TPC and were far away from the y-axis, suggesting that these components were the main source of the difference in LRF dried by different methods. According to the score plot of PCA analysis (Figure 4A), combined with the loading plot, the phytochemicals characteristic of LRF in FD were low moisture content and high TPC, Asn, PAC, and TAC content. Sun drying was opposite from FD. The phytochemicals characteristic of LRF in HD were high TPC and low TAC content. Based on the comprehensive principal component values (*F*) were calculated according to the formula: *F* = 0.595 × *F*_1_ + 0.396 × *F*_2_. From the results summarized in Table 2, the *F*-values of the phytochemicals content and antioxidant activity of LRF dried by different methods was in the order of FD > HD > SD. The result indicated that the quality of LRF in FD was optimal, followed by HD.

**Table 2:** The comprehensive principal component values (*F*) of LRF dried by different methods.

**No**.	**F1**	**F2**	**F**	**Rank**
FD3	4.2112	−0.8197	2.2086	1
FD2	4.0695	−1.3164	1.9523	2
FD1	2.3144	−0.9969	1.0242	3
HD2	−0.0217	2.8031	0.9598	4
HD1	−0.6780	2.9815	0.6332	5
HD3	−0.9524	2.9959	0.4757	6
SD2	−2.2563	−2.4244	−2.1770	7
SD3	−2.9536	−1.8616	−2.3945	8
SD1	−3.7330	−1.3616	−2.6824	9

### Effect of Drying Methods on Color and Antioxidant Activity of LRF Water Extraction

**Figure 5:**
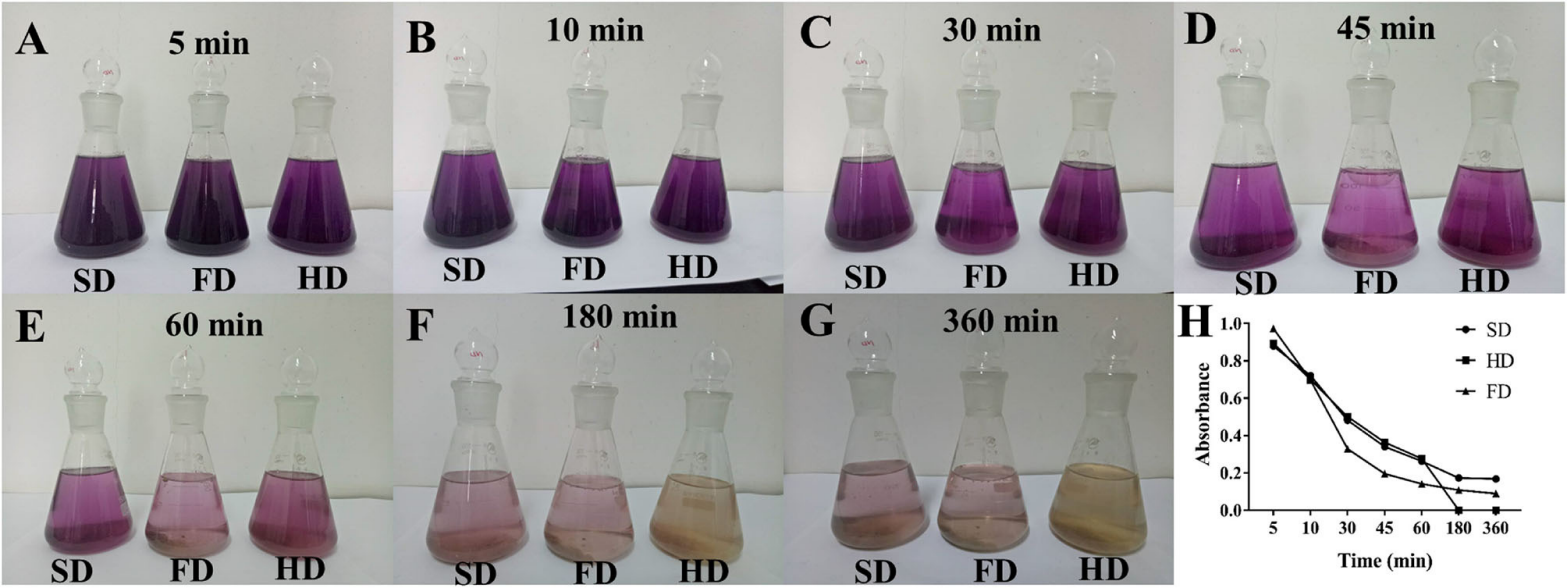
The water color of LRF dried by different methods **(A–G)** and the absorbance of the water color **(H)** at different soaking times.

Soaking of dried LRF in water and drinking has been one of the most used edible methods. The study found that different soaking times could affect the water color of LRF dried by different methods (Figures 5A–G). Different color of the water indicated different ingredients and chemical properties (55). To indicate the change of color, the full wavelength spectrum of the LRF water soaking was performed based on the method of UV-Vis. The results showed that the maximum absorbance was at 538 nm. It was in accordance with the absorbance (500–550 nm) of anthocyanin (26, 56), which illustrated that the values of absorbance could represent the color of the water. Thus, the absorbance of the soaking LRF water was detected; the results are shown in Figure 5H. The water color of LRF dried by different methods soaking for different times was different. The FD was the highest, and the color sharply dropped after 10 min. For HD, the color sharply dropped after 60 min. For SD, the color gently decreased. All three tests showed that the absorbance was lower after 60 min. These findings may be caused by the structure of LRF dried by three methods and the properties of anthocyanin. *Lycium ruthenicum* dried by FD was expansion and fragile, which promoted the dissolution rate of anthocyanin in water. However, anthocyanin was unstable in water and sharply decreased. For SD and HD, LRF was shrunken and hard. Anthocyanin dissolved slowly and decreased slowly, too.

**Figure 6:**
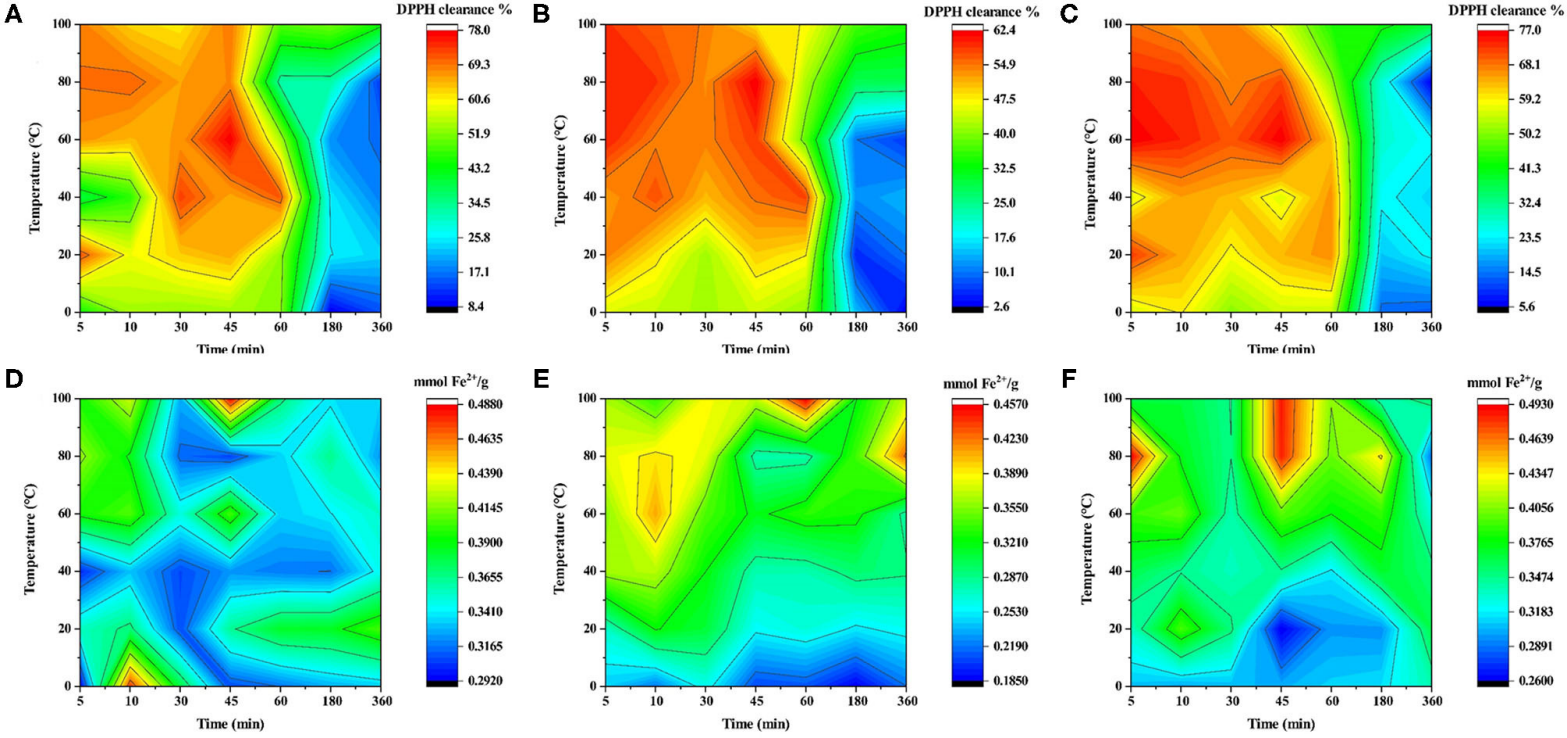
The color fill of antioxidant activity of LRF water soaking with different water temperatures and times. (From left to right: SD, HD, and FD; **A–C**: DPPH; **D–F**: FRAP).

The above study showed that soaking time had a different effect on the anthocyanin of LRF dried by different methods. Anthocyanin has antioxidant activity (26). To indicate that water temperature and soaking time had different antioxidant activity effect on LRF dried by different methods, DPPH and FRAP assay were performed and the results are shown in Figure 6. For DPPH (Figures 6A–C), water of LRF dried by three methods had a low clearance rate after 60 min and was not affected by the change in water temperature. From 5 to 60 min, the water temperature had an obvious effect on the clearance rate of DPPH. When soaking time was 5–50 min and temperature was 50–100°C, LRF dried by FD had a high antioxidant activity. For HD, high antioxidant active of DPPH was observed in two situations. The first was the higher water temperature (50–100°C) with a shorter soaking time (5–20 min). The second was the lower water temperature (40–80°C) with a longer soaking time (30–60 min). For SD, high antioxidant active of DPPH only was observed under the latter conditions. For FRAP (Figures 6D–F), when temperature is lower than 30°C, the water of LRF dried by FD had obvious low values of FRAP, and the high values were found at 70–100°C water temperature with 40–60 min soaking time. For HD, the high values of FRAP were considered at 50–80°C water temperature with 5–20 min soaking time. For SD, the high values of FRAP were considered at 60–100°C water temperature with 5–20 min soaking time. These findings may be caused by the different phytochemicals causing different reactions with DPPH and FRAP (54).

## Conclusion

This is the first extensive study on the influence that drying methods have on the quality of LRF. The appearance characteristic, moisture content, TPC, TFC, PAC, TAC, amino acid content, and antioxidant activity of LRF dried by FD, HD, and SD were all different. LRF dried by FD was round, expansive, and fragile, and maintained a larger amount of appearance traits. For SD and HD, the fruit exhibited shrinkage and hardness, especially for SD. PAC, Asn, TPC, TAC, and moisture content were the main sources of the difference in LRF dried by different methods. The phytochemical characteristics of LRF in FD were low moisture content and high TPC, Asn, PAC, and TAC content. Sun drying was opposite from FD. Hot air drying was high TPC and low TAC content. A comprehensive evaluation of the phytochemical component content and antioxidant capacity of LRF dried by the three different methods showed that the quality of LRF was in the order of FD > HD > SD. Additionally, results indicated that the change rate of anthocyanin in LRF dried by different methods with different soaking times was different. The color sharply dropped after 10 min for FD. For HD, the sharply dropped time delayed to after 60 min. In addition, the color gently decreased in SD. After 60 min, the color nearly faded. Based on the above finding, the antioxidant activity of LRF water at different water temperatures and soaking times was assayed, and the results showed that water temperature and soaking time had different antioxidant activity effects on LRF dried by different methods.

## Data Availability Statement

The original contributions presented in the study are included in the article/Supplementary Material, further inquiries can be directed to the corresponding author/s.

## Author Contributions

YL, ZQ, LJ, and HW designed the study. YL, XK, and JZ performed the laboratory work. YL and HW analyzed the data and wrote the paper. CG provided the fresh LRF samples. All authors contributed to the article and approved the submitted version.

## Funding

This study was supported by Research Project about Annual Medical Advantage Subject Group Construction in 2018 (XY201816), Special Talent Start-up Project (XT2020023), key research and development projects of Ningxia (2021BEG02040), Natural Science Foundation of Ningxia (2018AAC02009), Dao-di Herbs Eco-planting and Safeguard Project [Chinese traditional medicine science and technology (2020) No. 153], and Opening Subject of Key Laboratory of Pharmaceutical Creation and Generic Drugs in Ningxia.

## Conflict of Interest

CG was employed by company Ningxia Super-Kernel Health Management Technology Co., Ltd. The remaining authors declare that the research was conducted in the absence of any commercial or financial relationships that could be construed as a potential conflict of interest.

## Publisher's Note

All claims expressed in this article are solely those of the authors and do not necessarily represent those of their affiliated organizations, or those of the publisher, the editors and the reviewers. Any product that may be evaluated in this article, or claim that may be made by its manufacturer, is not guaranteed or endorsed by the publisher.
