# Supplementary material for: Composition Changes in Lycium ruthenicum Fruit Dried by Different Methods
Source: Front Nutr. 2021 Oct 5;8:737521. doi: 10.3389/fnut.2021.737521 (PMC8523835; doi:10.3389/fnut.2021.737521)
Supplement: Supplementary file 1 [file Data_Sheet_1.docx]

Supplementary Material

Composition Changes in *Lycium ruthenicum* Fruit

Dried by Different Methods

Youyuan Lu ^1,2^, Xiangfeng Kong ^1^, Juanhong Zhang ^1^, Chao Guo ^6^, Zhuo Qu ^1*^, Ling Jin ^4,5*^, Hanqing Wang ^1,2,3*^

^1^College of Pharmacy, Ningxia Medical University, Yinchuan, China

^2^Ningxia Engineering and Technology Research Center for Modernization of Regional Characteristic Traditional Chinese Medicine, Ningxia Medical University, Yinchuan, China

^3^Key Laboratory of Hui Ethnic Medicine Modernization, Ministry of Education, Ningxia Medical University, Yinchuan, China

^4^ School of Pharmacy, Gansu University of Chinese Medicine, Lanzhou, China

^5^ Northwest Collaborative Innovation Center for Traditional Chinese Medicine, Lanzhou, China

^6^ Ningxia Super-kernel Health Management Technology Co., Ltd, Yinchuan, China

*** Correspondence:**Hanqing Wang
[wwwhhq@163.com](mailto:wwwhhq@163.com)

Ling Jin
[zyxyjl@163.com](mailto:zyxyjl@163.com)

Zhuo Qu
quzhuo2008@163.com

**Table S1** Precursor/Product ion pairs and parameters for MRM of compounds used in this study

| Analytes | Retention time  (min) | MRM transitions  (Precursor→product) | Cone voltage  (V) | Collision energy  (eV) | [M+H]^+^ (m/z) |
| --- | --- | --- | --- | --- | --- |
| Pro | 5.88 | 116.23→70.22 | 34 | 14 | 116.23 |
| Leu | 4.76 | 132.23→86.28 | 26 | 8 | 132.23 |
| Arg | 8.92 | 175.30→70.22 | 30 | 18 | 175.3 |
| Asn | 7.45 | 132.88→73.85 | 2 | 12 | 132.88 |
| Asp | 7.00 | 134.20→74.19 | 24 | 12 | 134.2 |
| Lys | 9.04 | 147.30→84.20 | 24 | 14 | 147.3 |
| Tyr | 5.81 | 182.21→136.20 | 28 | 16 | 182.21 |
| Gln | 7.27 | 147.23→84.19 | 24 | 18 | 147.23 |
| Phe | 4.93 | 166.23→120.22 | 26 | 16 | 166.23 |
| Glu | 6.76 | 148.20→84.19 | 26 | 16 | 148.2 |
| Thr | 6.88 | 120.27→74.25 | 20 | 10 | 120.27 |
| Ser | 7.31 | 106.20→60.21 | 24 | 10 | 106.2 |
| Ile | 4.71 | 132.23→86.30 | 24 | 8 | 132.23 |
| His | 8.95 | 156.29→110.21 | 30 | 16 | 156.29 |
| Orn | 9.20 | 113.21→70.22 | 22 | 14 | 113.21 |
| Val | 5.48 | 118.23→72.23 | 22 | 10 | 118.23 |
| Cit | 7.51 | 176.23→113.25 | 22 | 16 | 176.23 |
| Met | 5.44 | 150.23→104.22 | 20 | 12 | 150.23 |
| Try | 4.95 | 205.23→146.21 | 26 | 20 | 205.23 |

**Table S2** Calibration curves, LOD and LOQ, recovery, precision, repeatability and stability of investigated compounds by UPLC-MS/MS

| Analytes | Calibration curves | r^2^ | Linear range (μg/mL) | LOD  (ng /mL) | LOQ  (ng /mL) | Recovery (%, n=3) | | Precision (RSD %) | | Repeatability  (RSD %, n=6) | Stability  (RSD %, n=6) |
| --- | --- | --- | --- | --- | --- | --- | --- | --- | --- | --- | --- |
|  |  |  |  |  |  | mean | RSD | Intraday (n=6) | Interday (n=6) |  |  |
| Pro | y=379330x+2553.10 | 0.9992 | 0.02-1.65 | 8.00 | 16.50 | 96.2 | 2.86 | 1.53 | 1.67 | 3.11 | 2.66 |
| Leu | y=320553x+3961 | 0.9995 | 0.02-1.79 | 6.00 | 18.00 | 97.1 | 2.32 | 0.98 | 2.43 | 1.32 | 1.26 |
| Arg | y=27836x+756.90 | 0.9997 | 0.04-2.96 | 16.20 | 32.50 | 99.6 | 4.14 | 2.89 | 3.01 | 3.11 | 3.07 |
| Asn | y=51867x+857 | 0.9955 | 0.02-1.96 | 6.50 | 19.60 | 96.5 | 3.96 | 2.11 | 3.01 | 3.24 | 4.67 |
| Asp | y=26289x+2523 | 0.9997 | 0.02-1.81 | 6.30 | 18.10 | 94.1 | 5.55 | 3.70 | 3.23 | 5.03 | 5.24 |
| Lys | y=231055x+3991.60 | 0.9998 | 0.02-1.66 | 6.50 | 16.60 | 101.5 | 3.75 | 3.66 | 5.39 | 4.44 | 4.65 |
| Tyr | y=96061x+1314 | 0.9998 | 0.02-1.67 | 8.10 | 16.70 | 96.1 | 3.86 | 1.21 | 2.94 | 4.29 | 3.46 |
| Gln | y=85107x+479.42 | 0.9999 | 0.02-1.97 | 7.60 | 19.70 | 94.2 | 5.29 | 3.99 | 5.02 | 5.11 | 3.31 |
| Phe | y=549448x-401.41 | 0.9977 | 0.02-2.36 | 7.90 | 23.60 | 102.8 | 3.66 | 1.23 | 2.98 | 1.78 | 1.71 |
| Glu | y=163246x+6811.60 | 0.9995 | 0.01-1.43 | 5.60 | 14.00 | 95.1 | 4.33 | 3.11 | 3.96 | 4.62 | 3.77 |
| Thr | y=21067x+306.66 | 0.9998 | 0.02-1.71 | 6.40 | 17.10 | 95.6 | 4.59 | 3.49 | 4.36 | 4.31 | 5.64 |
| Ser | y=10481x+787.26 | 0.9999 | 0.03-1.53 | 15.30 | 30.50 | 97.1 | 3.69 | 1.93 | 3.01 | 2.11 | 2.09 |
| Ile | y=231176x+3500.30 | 0.9995 | 0.02-1.74 | 7.60 | 17.40 | 98.9 | 2.38 | 0.93 | 2.03 | 2.68 | 2.71 |
| His | y=613549x+19448 | 0.998 | 0.02-1.58 | 5.30 | 15.80 | 95.7 | 3.86 | 1.31 | 2.95 | 1.79 | 2.38 |
| Orn | y=41229x+2743.40 | 0.9986 | 0.01-1.41 | 6.50 | 14.10 | 96.8 | 4.26 | 0.91 | 3.61 | 2.33 | 4.13 |
| Val | y=40000x+728.22 | 0.9998 | 0.02-1.61 | 6.50 | 16.10 | 96.4 | 4.75 | 1.11 | 2.55 | 3.78 | 3.17 |
| Cit | y=65204x-332.97 | 0.9998 | 0.02-2.03 | 10.10 | 20.20 | 97.2 | 2.69 | 0.69 | 1.81 | 5.01 | 4.94 |
| Met | y=47628x-278.31 | 1.0000 | 0.02-1.72 | 5.70 | 17.20 | 98.5 | 2.60 | 1.34 | 3.77 | 4.03 | 3.97 |
| Try | y=142061x-662.91 | 0.9992 | 0.02-1.60 | 5.30 | 16.00 | 98.6 | 3.02 | 0.73 | 3.83 | 3.43 | 1.99 |


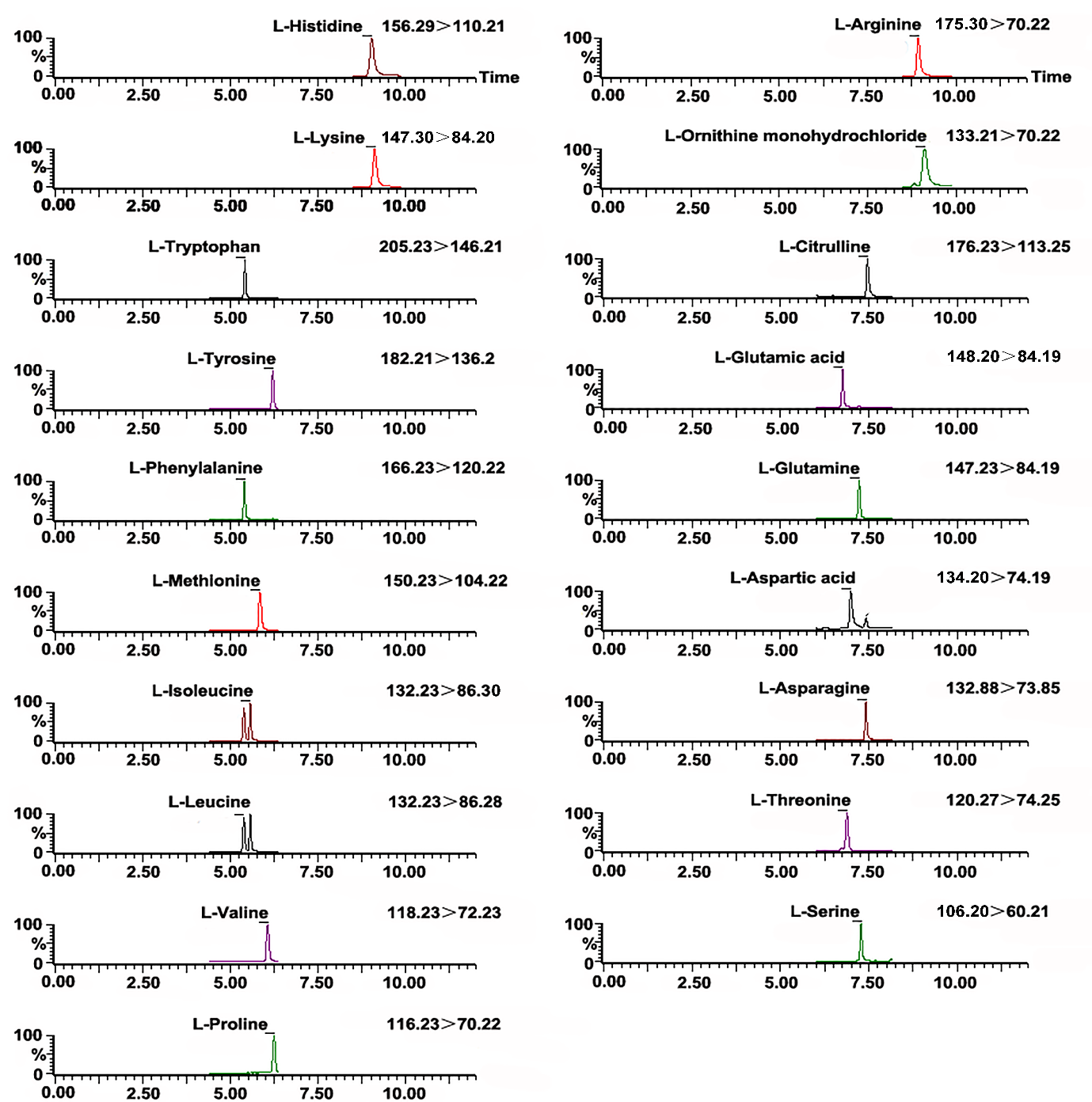


**Figure S1** Ultra-high-performance liquid chromatography coupled with tandem mass spectrometry (UHPLC-MS/MS) chromatography of mix standards for amino acids analyzed in this study.
